# Care of Psychiatric Patients: The Challenge to Emergency Physicians

**DOI:** 10.5811/westjem.2016.1.29648

**Published:** 2016-03-02

**Authors:** Leslie Zun

**Affiliations:** Mount Sinai Hospital, Department of Emergency Medicine, Chicago, Illinois

Psychiatric patients frequently present to the emergency department (ED) for care when they are in crisis. Recent studies demonstrate about 10% of all ED patients present with psychiatric illness.^1^ However, this is not an adequate estimate of the number of patients because many of these patients do not have a psychiatric diagnosis. Two recent studies have demonstrated that 45% of adults and 40% of pediatric patients who present to the ED with non-psychiatric complaints have undiagnosed mental illness.^2^–^3^ These studies did not determine whether these psychiatric illnesses affected the patients’ presentation. The purpose of this article is to discuss disparity and challenges in caring for these patients.

Psychiatric patients who present to the ED are just like other patients, or are they? For what other patient types do we have consultants outside of our medical facility come to evaluate the patient and determine the need for admission? For what other patient types are emergency physicians (EPs) uncomfortable ordering their home medications? What other patient types have to wait for an inpatient bed or to be transferred to another facility without receiving any treatment? What other patient type do our attitudes affect their outcome so significantly? Why do we focus on improving care for trauma, cardiac, stroke, pediatric, geriatric patients but not psychiatric ones? Is this an issue of benign neglect, lack of outcome satisfaction, unpleasantness of these patients, countertransference issues or something else that compels us to limit our interest in patient care, research, and learning more about these patients.

EDs do a good job of determining how to improve the care of the medical patient but they have done little addressing the unique needs of the psychiatric patients. Patient care surveys usually focus on evaluating the patient care experience of non-psychiatric patients in the ED. These customer service surveys have identified many priorities for patient care and satisfaction in the ED, need for improvement, and, for some physicians, determines a component of their bonuses. However, psychiatric patients have a unique set of preferences that differ from the non-psychiatric patients. They want verbal interventions, use of oral medications, input regarding their medication experiences and preferences, peer support services, improved discharge planning, a better triage process, reduced wait time for treatment and more privacy.^4^

EPs frequently have negative attitudes towards patients seen in the ED but it is most pronounced for psychiatric patients. They do not realize that their attitudes towards the psychiatric patient may lead to poor patient outcomes. It is noted that patients with intentional self-harm and substance use/abuse pose the most challenge for EDs.^5^ Stefan noted that emergency care providers regard psychiatric patients as problems or nuisances.^6^ There are a number of contributing factors to these findings including societal attitudes and personal biases, inadequate educational preparation, organizational climate, safety concerns, crowding, caregiver lack of confidence, and lack or guidelines.^6^ Although not studied in EDs, studies have noted that suicidal behavior appears to elicit mostly negative feelings among staff members.^7^ If not acknowledged and properly handled, these attitudes may lead to premature discharge. ED staff need to understand, contain and work through their negative feelings towards patients.

EPs have little training in behavioral emergencies in emergency medicine (EM) residencies. Education and experience is a problem when few EM programs provide experience or training in emergency psychiatry. This deficit is compounded by the fact that The American Board of Emergency Medicine board certification exam has 4% or less of the questions pertain to behavioral issues.^8^ EPs complain about gaps in detecting patients with substance use disorder, lack of education in care of psychiatric patients and a shortage of services to treat these patients.^6^ Nurses have issues with these patients as well. Nurses perceive lack of knowledge, skills and expertise, problems with triage risk assessment, frustration with frequent psychiatric patient visits, insufficient resources, ongoing patient and staff safety concerns, feeling of helplessness and perception of a broken mental health system.^6^

How do we resolve these challenges? We need more staff education and experience, care standards, better triage process, improved evaluation, enhanced treatment protocols and reduced wait time and boarding. There is a need for improved education and experience for psychiatric patients in EM. There are post-graduate fellowship programs in pediatric, geriatric, and critical care patients but few, if any, current fellowships in emergency psychiatry. Few programs have EM residents rotate on the consultation liaison psychiatric service or in a psychiatric ED. The solution to this deficit is to put a greater emphasis on psychiatric emergencies in residency, more questions on the EM board exam, and provide continuing medical education (CME) courses on psychiatric emergencies. Although many have expressed concern with “merit badges,” is it time to have a course for psychiatric emergencies like advanced trauma life support (ATLS), advanced cariovascular life support (ACLS), and pediatric advanced life support (PALS)? Whether there is a merit badge or not, there is a need for standardization in the care for patients with psychiatric emergencies. There is a need for a similar endeavor in the evaluation and treatment of the suicidal, depressed, psychotic or bipolar patient.

As we know, the role of the EP is to determine if the patient has a life- or limb-threatening problem, and to treat the acute symptoms and signs. The psychiatric patient has every bit the risk of some of our sickest medical patients. EPs must identify medical problems that mimic psychiatric illness, including metabolic, endocrine, infectious or substance induced that can put the patient’s life in immediate jeopardy. Furthermore, psychiatric patients are at some or even high risk for homicide, suicide or inability to perform self-care. Therefore, it is not an appropriate rationalization that psychiatric patients are any less deserving of a complete and thoughtful evaluation.

The triage process in the ED is skewed to patients with medical problems over those with psychiatric problems. Emergency Severity Index (ESI) triage system published by Agency for Health Quality Research (AHQR) is a five-tiered system that prioritizes patients presenting primarily with medical complaints, but is weak for triage of psychiatric patients in the ED. The Australian Triage Scale (Figure 1) and the Canadian Emergency Department Triage system for psychiatric patients are better tools, since they focus on properly assessing psychiatric patients.^9^ The Australasian mental health triage scale not only determines priority based on behavioral presentation but also places time parameters on the evaluation of a mental health patient. The ESI triage tool needs revision to reflect the needs of the psychiatric patient or adoption of another tool needs to be considered.

The evaluation process for psychiatric patients in the ED is problematic. Many EPs do not think that there is a need to perform a psychiatric evaluation. Not only is there a cost for an outside service to evaluate these patients, but also these do not eliminate a physician’s responsibility for appropriate disposition or reduce their liability exposure. EPs may not be able to continue to delegate their psychiatric evaluation to an outside source. Market forces from Accountable Care Organizations (ACO) to limit costs and admissions may force EPs to do this. Unfortunately for some, the ACOs may also determine need for admission.

A psychiatric evaluation is not as daunting as one might think. To differentiate medical illness or medical mimics from a psychiatric illness, an appropriate history, physical examination, mental status examination and clinically indicated testing are used. The medical clearance checklist (Figure 2) is one means to systematically perform and document this process. The other part of the psychiatric evaluation is determination of the need for a psychiatric inpatient admission. Unlike other medical illness this is not an exact science. Rather, diligence and good clinical judgement are the most valuable tools. Patients who have suicidal ideation need a risk assessment based on dynamic and static risk factors and protective components. The patient is placed into one of three risk categories: low (can be discharged home); moderate/(needs further psychiatric assessment); and high (inpatient psychiatric admission indicated).

A similar risk assessment is done for homicidal patients, which includes prior history, collateral information, means and a plan. It is easier to determine the patient’s ability to care for himself based on his clinical condition and ability to provide for his own needs. This assessment is performed by understanding insight and judgment of their mental illness and their ability to self-care for both medical and psychiatric illnesses.

Better treatments protocols are needed for psychiatric patients in crisis in the ED. All too frequently, these patients are given the same medication for agitation regardless of cause. However, best practices in evaluation and treatment of agitation (BETA) expert guidance recommends that medication be determined by the most probable etiology.^10^ We no longer treat all pneumonia patients with the same antibiotic, but assess their most likely etiology. Treatment of psychiatric illness should be similarly tailored to the patient and situation as detailed in Figure 3.

Boarding is more frequently seen in psychiatric patients than medical patients.^11^ This is primarily related to inadequate inpatient beds and outpatient resources. The boarding problem leads to iatrogenic worsening of their clinical condition and agitation. EPs need to explore alternatives to admission, rapid treatment and stabilization protocols, placement in a crisis stabilization unit and greater use of community resources.

EM has advocated for the care of the myocardial infarction, trauma, septic, pediatric and geriatric patients. It is time to advocate for the psychiatrically ill patient in the ED. We need to push for more training, establishment of standards of care, reduced wait times and find alternatives to boarding.

## Figures and Tables

**Figure 1:**
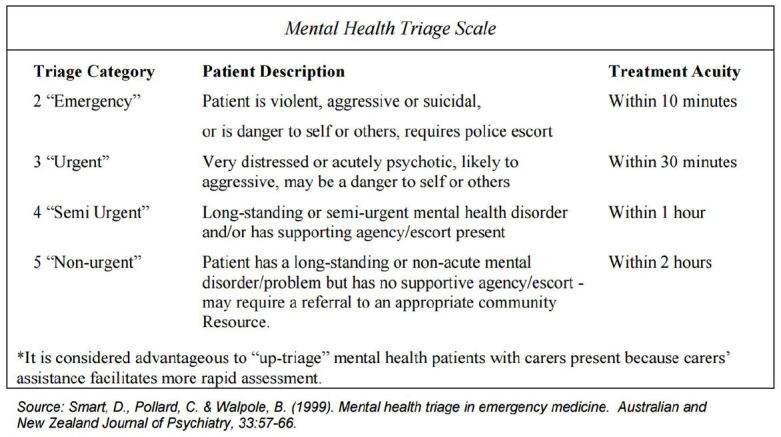
Australian mental health triage scale.

**Figure 2:**
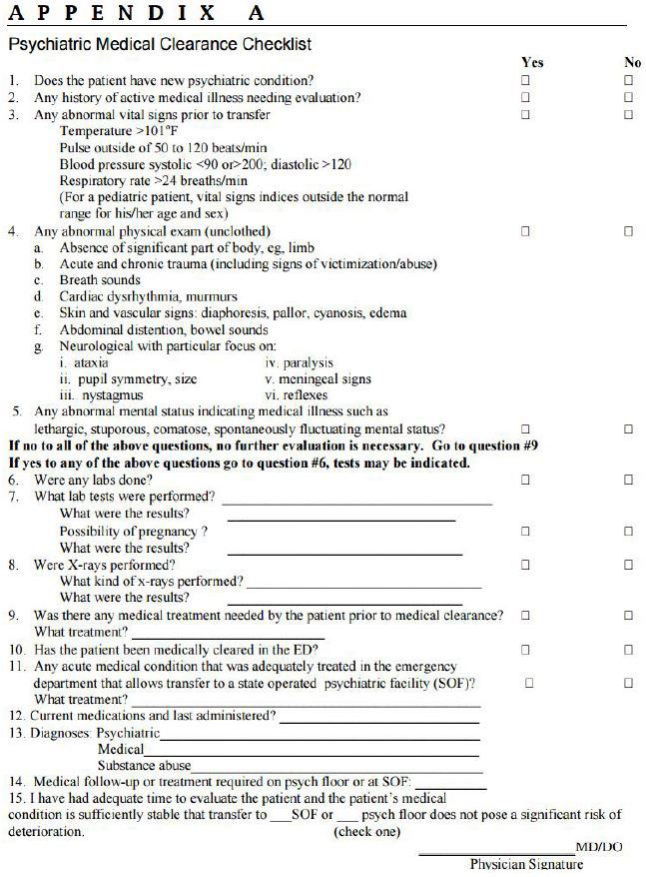
Psychiatric medical clearance checklist. *ED,* emergency department

**Figure 3:**
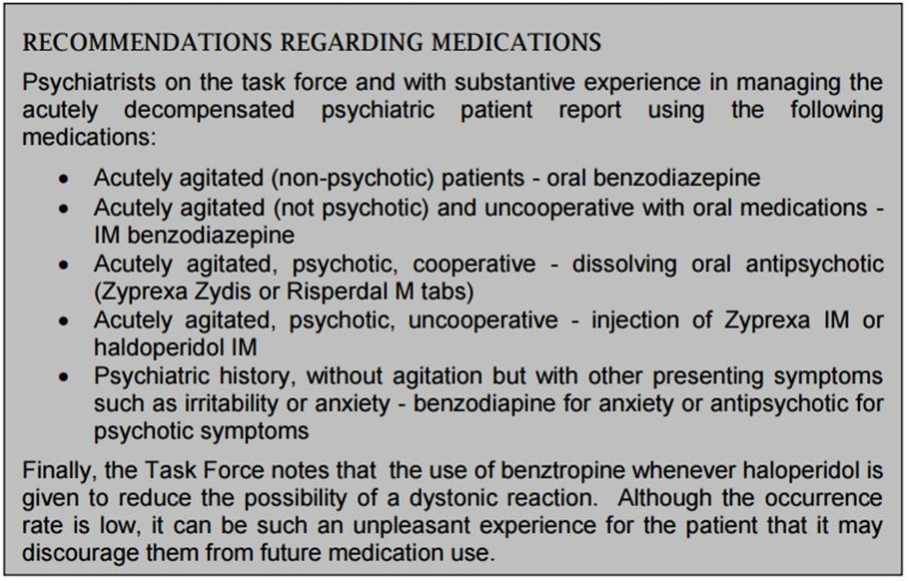
Recommendations for acute treatment of emergency department patients with agitation.
